# Chasing the Effects of Pre-Analytical Confounders – A Multicenter Study on CSF-AD Biomarkers

**DOI:** 10.3389/fneur.2015.00153

**Published:** 2015-07-08

**Authors:** Maria João Leitão, Inês Baldeiras, Sanna-Kaisa Herukka, Maria Pikkarainen, Ville Leinonen, Anja Hviid Simonsen, Armand Perret-Liaudet, Anthony Fourier, Isabelle Quadrio, Pedro Mota Veiga, Catarina Resende de Oliveira

**Affiliations:** ^1^Center for Neuroscience and Cell Biology (CNC), University of Coimbra, Coimbra, Portugal; ^2^Neurochemistry Laboratory, Neurology Department, Coimbra University Hospital, Coimbra, Portugal; ^3^Faculty of Medicine, University of Coimbra, Coimbra, Portugal; ^4^Neurology Department, Institute of Clinical Medicine, Kuopio University Hospital, University of Eastern Finland, Kuopio, Finland; ^5^Neurosurgery of NeuroCenter, Kuopio University Hospital, Kuopio, Finland; ^6^Danish Dementia Research Centre, Copenhagen University Hospital Rigshospitalet, Copenhagen, Denmark; ^7^Neurobiology, Biochemistry and Molecular Biology Department, University Hospital of Lyon, Lyon, France; ^8^UMR5292, BioRan, CNRS, INSERM U1028, University of Lyon 1, Lyon, France; ^9^Société Française de Biologie Clinique (SFBC), Alzheimer Biomarkers Group Co-Coordination, Lyon, France; ^10^Statistics and Research – Curva de Gauss, Training and Consulting, Canas de Senhorim, Portugal

**Keywords:** Alzheimer’s disease, cerebrospinal fluid, biomarkers, BIOMARKAPD, standardized operating procedures, β-amyloid, tau protein, phosphorylated tau protein

## Abstract

**Introduction:**

Core cerebrospinal fluid (CSF) biomarkers – Aβ42, Tau, and phosphorylated Tau (pTau) – have been recently incorporated in the revised criteria for Alzheimer’s disease (AD). However, their widespread clinical application lacks standardization. Pre-analytical sample handling and storage play an important role in the reliable measurement of these biomarkers across laboratories.

**Aim:**

In this study, we aim to surpass the efforts from previous studies, by employing a multicenter approach to assess the impact of less studied CSF pre-analytical confounders in AD-biomarkers quantification.

**Methods:**

Four different centers participated in this study and followed the same established protocol. CSF samples were analyzed for three biomarkers (Aβ42, Tau, and pTau) and tested for different spinning conditions [temperature: room temperature (RT) vs. 4°C; speed: 500 vs. 2000 vs. 3000 g], storage volume variations (25, 50, and 75% of tube total volume), as well as freezing-thaw cycles (up to five cycles). The influence of sample routine parameters, inter-center variability, and relative value of each biomarker (reported as normal/abnormal) was analyzed.

**Results:**

Centrifugation conditions did not influence biomarkers levels, except for samples with a high CSF total protein content, where either non-centrifugation or centrifugation at RT, compared to 4°C, led to higher Aβ42 levels. Reducing CSF storage volume from 75 to 50% of total tube capacity decreased Aβ42 concentration (within analytical CV of the assay), whereas no change in Tau or pTau was observed. Moreover, the concentration of Tau and pTau appears to be stable up to five freeze–thaw cycles, whereas Aβ42 levels decrease if CSF is freeze-thawed more than three times.

**Conclusion:**

This systematic study reinforces the need for CSF centrifugation at 4°C prior to storage and highlights the influence of storage conditions in Aβ42 levels. This study contributes to the establishment of harmonized standard operating procedures that will help reducing inter-lab variability of CSF-AD biomarkers evaluation.

## Introduction

Cerebrospinal fluid (CSF) biomarkers, Aβ42, Tau protein, and phosphorylated Tau (pTau), are frequently assessed for their proven value as hallmarks of initial and coursing neuropathological events in Alzheimer’s disease (AD) (1, 2). Studies over the years have shown a 250–300% increase of CSF Tau and pTau and a decrease of about 50% in CSF Aβ42 in AD patients compared to normal aging (3). The high sensitivity and specificity of these markers have been shown to be useful in discriminating AD from other dementias, as well as to identify AD before onset of dementia (the stage known as mild cognitive impairment – MCI), both in single-center and large-scale multicenter studies (4–7). Therefore, biomarkers have recently been incorporated in the new proposed revised criteria for AD (8). The development and application of revised diagnostic criteria, which include biomarkers, will substantially improve the diagnostic accuracy for AD toward other forms of dementia and can help anticipate the rate of progression and early disablement in AD (9, 10). Besides giving clues to pathogenic mechanisms of the disease, biomarkers can also favor therapeutics development by signaling desired effects of drugs in phase I–II clinical trials, allowing inclusion of early cases to longitudinal studies and even identifying sub-groups of patients in order to tailor treatment (11, 12).

However, in recent years, international scientific evaluation studies regarding neurochemical diagnosis of neurodegenerative diseases have shown that the inter-laboratory precision of those biomarkers measurements requires optimization (13). Cut-offs differ greatly between studies, and the widespread clinical application of revised criteria for early AD is hampered by lack of standardization of biomarkers (2, 8). These variations in biomarkers performance can be the result of several pre-analytical and analytical factors. Pre-analytical factors include lumbar puncture (LP), CSF handling, and storage procedures, while analytical factors are more assay-related, for instance to differences among centers in training of technicians, operating procedures, or batch-to-batch variations of kits (14, 15). Analytical outcome can also be influenced by biological variables intrinsic to study participants, such as genetic variations or relation between CSF and brain volume (16).

Several international standardization initiatives are already ongoing. The most extensive is the global Alzheimer’s Association external quality control program for CSF measurements led by K. Blennow (17, 18), involving more than 80 laboratories worldwide. However, it is still purely descriptive and does not provide any active interventions to tackle variations. Among interested and connected centers for harmonization of AD biomarkers, there have been attempts to reach a consensus concerning CSF collection, handling, and storage, and to create uniformized standard operating procedures (USOPs) (19, 20). Reasonable amount of evidence already exists regarding how CSF biomarkers levels are influenced by certain pre-analytical conditions. For instance, it has been well-established that polypropylene (PP) tubes and pipet tips should be used for CSF collection, handling, and storage, since lipophilic proteins like Aβ peptides bind in a non-specific manner to non-PP tubes (21, 22). Several laboratories have also reported on the stability of CSF proteins between collection and storage (13, 23, 24), and it is a common consensus that they are stable for at least 5 days at 4°C (20). Moreover, some studies have analyzed the influence of freeze/thaw cycles on CSF-AD biomarkers and most have found a decrease in Aβ42 concentration as a result of freeze/thaw cycles, but different results were found in the number of cycles that led to this decrease.

However, all of these studies were done in a single center and employing a limited number of samples, generally no more than 10 samples per experimental condition. Also, most of them only address the effect of pre-analytical conditions on CSF Aβ42 and Tau levels or just in Aβ42, with only a few studies looking at the effect on pTau levels (13, 25). Therefore, a standardized protocol for handling CSF is still needed to allow for multicenter studies and data comparisons in a near future (18).

In 2011, a new consortium was launched under the scope of Joint Program for Neurodegenerative Diseases (JPND), the BIOMARKAPD, expected to exceed all ongoing initiatives as it involved a real European effort to solve standardization issues. The main aim of the project is to develop evidence-based guidelines for measurement and use of biomarkers in AD and Parkinson’s disease (PD) in clinical practice, within 48 sites from 21 European countries and also Canada. The present multicenter study is part of this transnational project and its main aim is to assess CSF pre-analytical confounding factors, which have been less studied so far, that can possibly affect assay performance and biomarkers measurements across laboratories. We intend to test a large number of samples, for all three biomarkers, the effect of different spinning CSF conditions (temperature and speed) and of storing different CSF volumes per total tube volume into aliquots. We will also extend the study of the impact of the number of freeze–thaw cycles (up to five cycles) to pTau. By this, we expect to contribute to the development of new feasible, CSF handling USOPs that will help reducing interlaboratory variability of CSF-AD biomarkers.

## Materials and Methods

### Participants

Four centers (Neurochemistry Laboratory, Coimbra University Hospital, Portugal; Institute of Clinical Medicine-Neurology, Kuopio University Hospital, Finland; Danish Dementia Research Centre, Copenhagen University Hospital Rigshospitalet, Copenhagen, Denmark; Neurobiologie, University of Lyon, Lyon, France) participated in this study. All laboratories handled CSF samples in a standardized way, through the same previously established protocol. Contributions in terms of number of samples were variable (see Table 1). This study has been approved by the Ethical board of Coimbra’s University Hospital, by local Ethical committee of French Ministry of Research and Higher Education, Ethical committee from the Capital Region of Denmark, and by Research Ethics Committee Hospital District of Northern Savo.

**Table 1:** **Participating centers and their sample contribution for the evaluation of CSF pre-analytical conditions (temperature and speed of centrifugation, CSF%/tube volume, and number of freeze/thaw cycles) on Aβ42, Tau, and pTau levels**.

	Centrifugation		%CSF/tube vol.	Freeze–thaw cycles
	Temperature	Speed	
Coimbra (Portugal)	27	22	30	27
Ctr = 0%; AD = 25.8%; MCI = 12.1%	
OD = 28.8%; OT = 33.3%	
Copenhagen (Denmark)	8	8	–	–
OT = 100%	
Kuopio (Finland)	10	–	10	10
Ctr = 10%; AD = 2.5%; MCI = 2.5%	
OD = 5%; OT = 80%	
Lyon (France)	10	10	10	3
Ctr = 4.5%; AD = 18.2%; MCI = 4.5%	
OD = 36.4%; OT = 13.6%	
TOTAL = 136	55	40	50	40

*Ctr, healthy controls; AD, Alzheimer’s disease; MCI, mild cognitive impairment; OD, other dementias; OT, other*.

### CSF collection

The study was performed with freshly collected CSF samples, obtained by LP in the L3/L4 or L4/L5 intervertebral space by clinicians in the Neurology Departments of each center, using a 20 or 25 G needle and collected to 10 mL standardized PP tubes (Sarstedt 62.610.201). A total of 136 samples were collected from patients with different diagnoses, five of them not classified (healthy controls – 3.7%; MCI – 7.4%; AD – 16.2%; other dementia – 21.3%; other diagnosis – 47.8%). A small amount of CSF was used for routine analysis including cytological (white and red cell count) and chemical analysis (total protein and glucose content). From a total of 133 patients with this information, 50.7% had normal RBC count and 47.1% abnormal count; for CSF total protein, 69.1% had normal (*n* = 88; 32.2 ± 6.9 mg/dL; 15–44 mg/dL) content and 27.9% abnormal (*n* = 44; 61.2 ± 14.3, 45–99 mg/dL). The remaining CSF was processed according to the different pre-analytical conditions to be tested further on. In all cases, samples were handled at room temperature (RT) (18–25°C), and exposure to light and time between CSF collection and storage did not exceed 2 h.

### Tested pre-analytical conditions

#### Centrifugation

For each sample, CSF was first aliquoted (380 μL) into five PP tubes of 500 μL (Sarstedt ref. 72.730.006). Tube C1 was not spinned at all and was left standing at RT without spinning until other tubes were ready (kept into an intermediate tube until transfer to final aliquot in order to keep the same procedure compared to other centrifuged conditions); Tube C2 was centrifuged for 10 min, 2000 × *g* at RT; Tube C3 was centrifuged for 10 min, 2000 × *g* but at 4°C (standard condition used for routine processing at all four centers); Tube C4 and C5 underwent spinning for 10 min at RT, the former at 500 × *g* and the latter at 3000 × *g*. Tubes C2 and C3 were used to test the effect of temperature during centrifugation and Tubes C2, C4, and C5 to test for speed. We also compared Tubes C1 (no spinning) and C3 (routine protocol). The supernatant of centrifuged CSF, as well as the non-centrifuged CSF, was then immediately transferred from spinning tubes to final set of tubes (500 μL Sarstedt ref. 72.730.006) and frozen at −80°C until analysis.

To test the impact of RBC count in CSF, five different samples were spiked with blood at 1/1000 (3.6 μL in 3.5 mL of CSF) to reach a final number of 5000 RBC/μL (±10%). The spiked CSF was aliquoted and treated as described above.

#### CSF%/Tube Volume

For each sample, CSF was first centrifuged for 10 min, 2000 × *g* at 4°C, and then aliquoted into tubes, as described above, in order to fill different percentages of total tube volume – V1 (25%; i.e., 500 μL in a 2 mL tube; Sarstedt ref. 72.694.007); V2 (50%; 250 μL in a 500 μL tube; this volume represents the minimum amount required to perform the assays for Aβ42, Tau, and pTau); V3 (75%, our baseline condition, i.e., 380 μL in a 500 μL tube). The aliquoted CSF was then immediately stored at −80°C until analysis.

#### Freeze/Thaw Cycles

To test this condition, we aliquoted the same volume (380 μL) of centrifuged CSF (10 min, 2000 × *g* at 4°C) into three 500 μL tubes and stored them at −80°C. One of them (F1, baseline condition) was left frozen until the moment of analysis; for tube F2, we forced two freeze–thaw cycles (left on the benchtop for 2 h at RT to mimick assay time on two consecutive days after collection) prior to analysis, which would account for a total of three cycles; for tube F3, four freeze/thaw cycles were done prior to the day of analysis, therefore reaching a total of five freeze/thaw cycles.

### CSF analysis

All samples were quantified within 1 month of storage at −80°C. CSF levels of Aβ42, total Tau, and pTau 181P were determined using commercially available single-analyte ELISA kits [INNOTEST^®^ β-AMYLOID (1–42), INNOTEST^®^ hTAU-Ag, and INNOTEST^®^ PHOSPHO-TAU (181P), Fujirebio, Spain], according to the manufacturer’s instructions and consensus practices from within BIOMARKAPD consortium. All samples were run in duplicate and all conditions tested for the same sample were run simultaneously on the same ELISA plate. Concentrations were extrapolated from a four-parameter Sigmoidal Curve. If the CV of duplicates was >20%, samples were excluded from the study to avoid additional confounding factors. If concentrations were below the limit of detection of the method, the value was set equal to the lowest standard of the calibration curve. None of the samples were above the concentration of the highest standard for each of the assays. Results were expressed in picogram per milliliter and as a relative percentage of the baseline conditions. All the participants in the study were asked to classify each sample as “normal” or “abnormal,” according to their own cut-off levels for Aβ42, Tau, and pTau.

### Statistical analysis

The statistical analysis was accomplished with SPSS for Windows version 22.0 and Graph Pad Prism 6.0. The following variables were tested for each protein assay (Aβ42, Tau, and pTau): centrifugation temperatures – “2000 × *g*/4°C” vs. “2000 × *g*/RT”; centrifugation speeds – “RT/500” vs. “RT/2000” vs. “RT/3000 × *g*,” and also “no spinning” vs. protocol (2000 × *g*/4°C); percentage of CSF per total tube volume – “25” vs. “50” vs. “75%”; for freeze/thaw cycles – “1” vs. “3 ” vs. “5 cycles”. *t*-test was used for pairwise comparisons. Repeated measures were first performed for multiple comparisons and also adding the following co-variates: “Center,” “Clinical Group,” “Biomarker classification” as normal or abnormal according to laboratory cut-offs, “CSF Total Protein,” and “RBC count” either as scale or ordinal variable. *Post hoc* tests (Bonferroni’s) were applied to repeated measures testing, when multiple comparisons were significant. Correlations between variables were performed using Pearson’s correlation coefficient. As only five samples were used for studying the effect of blood spiking in CSF, non-parametric tests for pairwise comparisons were used as Friedman and Wilcoxon.

## Results

### Influence of centrifugation parameters

We first analyzed the results obtained from non-centrifuged aliquots comparing to those centrifuged under protocol conditions (2000 × *g* at 4°C for 10 min). We observed no significant difference between protein concentrations and found that the absence of centrifugation seemed not to affect the outcome. Next, we looked for variations within spinning temperatures, 4°C (routine protocol) and RT. No significant difference was observed for any of the three biomarkers. When testing centrifugation speeds (500, 2000, and 3000 × *g*), still no statistically significant change was seen in any of the biomarkers (Table 2).

**Table 2:** **Concentration of each biomarker (picogram/milliliter) according to the three different pre-analytical confounders**.

Confounders	Biomarkers (pg/mL)		
	Aβ42	Tau	Phospho-Tau
**Centrifugation**			
Non-centrifuged	548.6 ± 233.9 (*n* = 55)	315.2 ± 194.8 (*n* = 55)	47.0 ± 25.0 (*n* = 55)
4°C	537.9 ± 224.7 (*n* = 55)	321.4 ± 197.4 (*n* = 55)	46.8 ± 25.1 (*n* = 55)
RT	524.3 ± 241.0 (*n* = 55)	316.1 ± 191.0 (*n* = 55)	47.3 ± 25.6 (*n* = 55)
500 × *g*	556.3 ± 230.1 (*n* = 40)	330.4 ± 211.6 (*n* = 40)	49.4 ± 27.9 (*n* = 40)
2000 × *g*	558.6 ± 239.7 (*n* = 40)	330.6 ± 209.0 (*n* = 40)	49.0 ± 27.6 (*n* = 40)
3000 × *g*	554.7 ± 234.0 (*n* = 40)	335.1 ± 216.2 (*n* = 40)	48.3 ± 26.4 (*n* = 40)
**% CSF/tube vol.**			
25	651.9 ± 337.1 (*n* = 50)	320.0 ± 214.9 (*n* = 50)	48.0 ± 26.9 (*n* = 50)
50	636.7 ± 352.0 (*n* = 50)*	323.4 ± 232. 3 (*n* = 50)	47.6 ± 25.8 (*n* = 50)
75	657.6 ± 334.4 (*n* = 50)	323.8 ± 230.1 (*n* = 50)	48.1 ± 26.1 (*n* = 50)
**Freeze–thaw cycles**			
1 time	597.9 ± 237.0 (*n* = 40)	353.9 ± 243.4 (*n* = 40)	47.5 ± 30.1 (*n* = 40)
3 times	597.5 ± 243.2 (*n* = 40)	358.2 ± 245.2 (*n* = 40)	47.5 ± 30.3 (*n* = 40)
5 times	569.8 ± 220.4 (*n* = 40)‡, 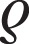	358.5 ± 247.1 (*n* = 40)	47.8 ± 30.5 (*n* = 40)

*Results are expressed in mean ± SD (95% CI)*.

*RT, room temperature; tube vol., tube volume*.

*Aβ42 %CSF/Tube vol: *p < 0.05 vs. 75%; Aβ42 Freeze/Thaw cycles: 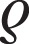
*p* = 0.072 vs. three times; ‡*p* < 0.05 vs. one time*.

*Centrifugation: *N* = 55-Ctr = 7.3%; AD = 18.2; MCI = 7.3%; OD = 23.6%; OT = 41.8%; *N* = 40-AD = 15%; MCI = 7.5%; OD = 30%; OT = 45%; %CSF/Tube Vol.: Ctr = 2.0%; AD = 18%; MCI = 10%; OD = 26%; OT = 36%; freeze–thaw cycles: AD = 25%; MCI = 10%; OD = 20%; OT = 45%*.

**Figure 1:**
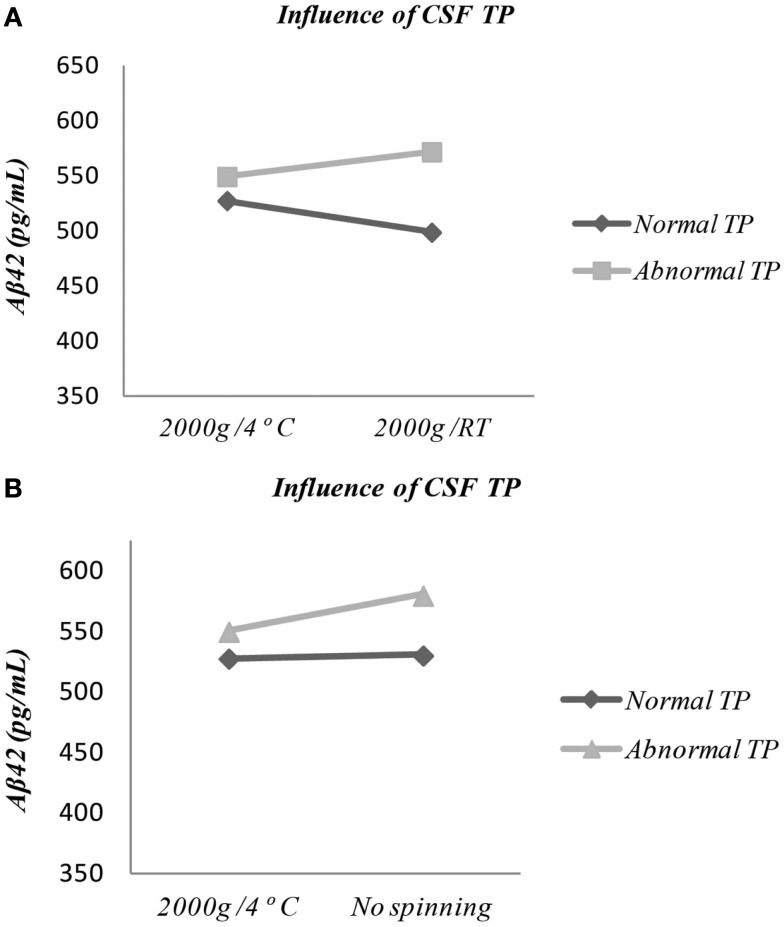
**(A)** Significant influence of covariate “CSF Total protein content” (dichotomized in normal/abnormal facing reference values) in the comparison between Aβ42 levels after centrifugation at 4°C vs. room temperature (*p* = 0.029) (TP, total protein; RT, room temperature). **(B)** Discrete influence of covariate “CSF Total protein content” (dichotomized in normal/abnormal facing reference values) in the comparison between Aβ42 levels after centrifugation at 2000 × *g*/4°C vs. no spinning at all (*p* = 0.176). TP, total protein; Absolute values – no spinning: normal TP – 529.7 ± 234.2, abnormal TP – 579.2 ± 249.2); 4°C: normal TP – 498.4 ± 237.2, abnormal TP – 549.4 ± 238.0.

Data were reanalyzed, testing the influence of the following co-variates: “Center,” “Clinical Group,” “CSF Total Protein,” and “RBC count” (both scale and dichotomized in normal/abnormal), “Biomarker Classification” in normal or abnormal for each protein according to each laboratory cut-offs. “CSF Total protein (TP),” dichotomized as normal/abnormal, influenced the effect of centrifugation conditions on Aβ42 levels (Figure 1A; *p* = 0.029). Samples with high TP (>44 mg/dL) had increased levels of Aβ42 if centrifuged at RT (571.5 ± 261.8) compared to 4°C (549.4 ± 238.0), whereas samples with normal TP had higher Aβ42 levels when centrifuged at 4°C (527.2 ± 226.2, 4°C vs. 498.4 ± 237.2, RT). Moreover, in samples with high TP content, which were not centrifuged, Aβ42 levels tended to increase in relation to centrifugation under baseline conditions (Figure 1B; *p* = 0.176). Other covariates had no impact concerning centrifugation conditions for the three markers.

Regarding experiments with blood spiked CSF, as we have tested only a limited number of samples, results are only indicative and could be used to define a more precise protocol. We observed no significant difference between protein concentrations using variations in spinning temperatures, 4°C (routine protocol) and RT. No significant difference was observed for any of the three biomarkers. When testing centrifugation speeds (500, 2000, and 3000 × *g*), again no statistically significant change was seen in any of the biomarkers. However, when we compared data obtained after centrifugation (routine protocol) and no centrifugation, we found a statistical increase of mean levels of Aβ42 and pTau in no centrifuged spiked samples by 6 and 11% (*p* < 0.05), whereas Tau levels were not impacted by the absence of centrifugation (data not shown).

### Influence of CSF percentage per total tube volume

**Figure 2:**
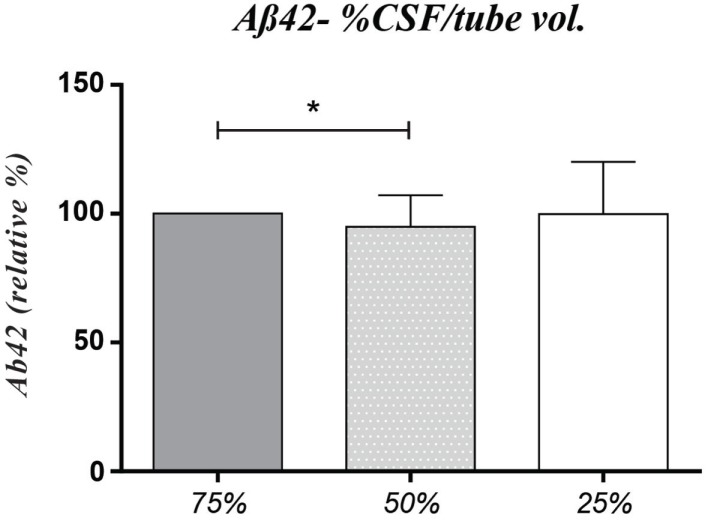
**Observed differences in Aβ42 levels between aliquots with 25 vs. 50 vs. 75% of total tube volume (75 vs. 50%, *p* = 0.03)**. Results expressed in relative percentage, facing the baseline condition (75% of tube volume representing 100%). Absolute concentration levels (mean ± SD): 25% – 651.9 ± 337.1; 50% – 636.7 ± 351.9; 75% – 657.6 ± 334.4.

**Figure 3:**
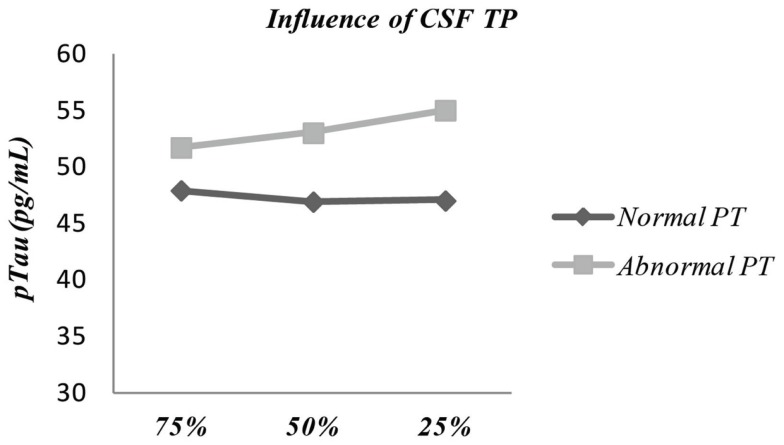
**Influence of covariate “CSF Total protein” (dichotomized in normal/abnormal facing reference values) in the comparison between pTau levels after aliquoting with different percentages of CSF per tube volume – 25 vs. 50 vs. 75% (*p* = 0.027)**. TP, total protein.

We hypothesized that the amount of CSF aliquoted in relation with total tube volume would have impact on protein concentration mainly because of the adhesive ability of Aβ42 and possibly Tau to tube walls, even though PP vials were always used throughout the study. Thus, different CSF volume percentages were tested in final aliquots and we found that decreasing the percentage of tube filling from 75% (baseline condition) to 50% resulted in a small but significant reduction of 3.7% in Aβ42 concentration (*p* = 0.03). This effect was indistinguishable from the analytical coefficient of variation of the assay. Moreover, when further decreasing the percentage of tube filling to 25%, Aβ42 levels increased to levels similar to the ones observed under baseline conditions (Figure 2). Neither Tau nor pTau proteins levels were influenced by the amount of CSF aliquoted in relation with total tube volume. Adding covariates to our tests showed influence of “CSF Total protein” (dichotomized as normal/abnormal) on pTau levels (*p* = 0.027), particularly in 25% filling volume aliquots presenting abnormal TP content (54.6 ± 30.5) vs. normal TP (47.1 ± 26.8) (Figure 3). Other covariates had no impact in any of the three biomarkers.

### Influence of number of freeze–thaw cycles

In this section, we tried to simulate the frequent real-life need of defrosting a sample for other purposes, prior to biomarker measurements. Therefore, we compared the results of a regular procedure, where the sample is just thawed for biomarker assessment (one cycle), with other possible situations (thawed for two and four times prior to protein assay).

**Figure 4:**
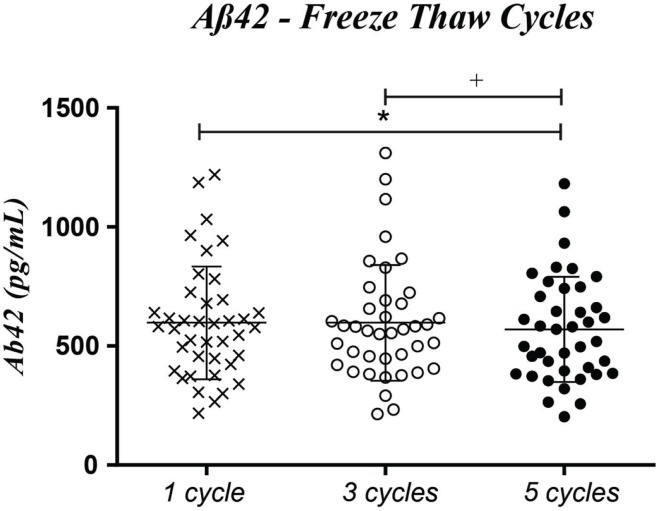
Observed differences in Aβ42 levels between samples freeze/thawed three and five times prior to the moment of analysis (1 cycle) (1 vs. 5 cycles, *p ***=*** 0.028; 3 vs. 5 cycles, ^+^*p* = 0.072). Absolute concentration levels (mean ± SD): one time – 597.9 ± 237.0; three times – 597.5 ± 243.2; five times – 569.7 ± 220.4.

We observed that while Tau and pTau remain stable for up to the five freeze/thaw cycles, the same is not true for Aβ42. Although thawing the CSF sample three times did not change the measured Aβ42 levels, a statistical significant reduction of 5.0% in Aβ42 levels was observed when the number of freeze–thaw cycles was increased to 5 (Figure 4; *p* = 0.028). However, this decrease remained in the analytical coefficient of variation of the assay. Covariate inclusion had no impact, concerning the effect of the number of freeze/thaw cycles, for the three markers.

As the presence of increased total CSF proteins was the only covariate influencing the results, we studied the correlation between the levels of each biomarker and CSF protein content. We found significant correlations (*p* < 0.05, data not shown) between pTau levels and CSF TP in both non-centrifuged and centrifuged samples under protocol conditions (2000 × *g*, 4°C).

## Discussion

There have been a few studies exploring potential CSF pre-analytical confounders, but a systematic analysis of some conditions, such as centrifugation, storage volumes, and freeze/thawing cycles, is still missing. To the best of our knowledge, our study has so far the largest number of samples (over 40 samples for each condition) testing systematically these three potential major sources of variability. This study includes samples from different cohorts of patients with several clinical diagnosis, validating our results for a broad range of biomarkers quantitative values. Furthermore, the influence of multiple co-variates was evaluated, and all other variables related to CSF collection were strictly controlled (19, 26). In this study, all LPs were performed in the morning, as although no clear diurnal pattern in Aβ42 levels has been observed, a 1.5- to 4-fold variation in AD biomarkers during a 36 h period has been reported (20). Moreover, fasting, as well as possible adsorption to lumbar catheter walls during LP, was also suspected to influence Aβ42 levels, but so far such effects could not be demonstrated (27). Current guidelines regarding collection and storage tubes (14) were also strictly followed and CSF was processed and frozen within a maximum of 2 h after collection. According to some reports, Aβ42 content is altered if CSF is not immediately frozen (to avoid protein oxidation), while Tau proteins are stable and can be kept at room temperature up to 24 h (25) or even 4 days (13). The use of different types of tube materials (polycarbonate, polystyrene, PP, and other copolymers) has also been tested. In several studies comparing PP against others plastics, authors never tested the variability among the different PP tubes, therefore remaining a potential confounder (22, 27). A few studies compared several PP tubes and concluded that PP is not a warranty against adsorption and only specific tubes reported to avoid adsorption could be recommended (21, 28, 29). A very recent study has shown that even using only PP tubes, Aβ42 levels are reduced up to 25% simply through multiple tube transferences which, therefore, should be minimized (28).

Speed and temperature of CSF centrifugation vary considerably between laboratories. Therefore, in this study, we tested both the influence of spinning temperature (RT and 4°C) and speed (500, 2000, and 3000 × *g*) on biomarkers levels. Overall, neither of these conditions were found to influence the concentration of any of the biomarkers. A previous study has looked for the influence of CSF centrifugation protocols on Aβ42 levels (27) and observed a significant decrease in Aβ42 concentration in centrifuged samples (10 min, 2000 × *g*, either at RT or 4°C) compared to non-centrifuged samples. It has also been found that no difference occurred in Aβ42 and Tau levels in CSF samples stored at 4°C and centrifuged after 1, 4, 48, or 72 h (30). Furthermore, no differences were found comparing centrifuged samples immediately frozen and those left for 4 days at 4°C without spinning (30). Centrifugation speed has been reported not to have an effect on biomarkers levels, but the centrifugation of hemorrhagic samples at 2000 × *g*, RT, within 2 h after collection, in order to avoid cell lysis, is recommended (26).

The inclusion of covariates in our analysis showed that, in samples with a high total protein content, an increase in Aβ42 concentration upon centrifugation at RT occurs. It can be hypothesized that Aβ42 can bind to excess protein, thus preventing the adhesion to tube walls, and this interaction may be disrupted by freeze/thawing. In contrast, in samples with normal or low TP content, centrifugation at RT may promote the adhesion of Aβ42 to tube walls, leading to lower measured levels of the peptide. As the largest discrepancy in Aβ42 levels between samples with normal/abnormal TP is seen when centrifugation is done at RT, spinning at 4°C should be applied routinely. In line with a previous study, we also observed that no spinning increases the measured levels of Aβ42 in samples with a high CSF total protein content (27). We cannot neglect another hypothesis, consistent with a competition between proteins present in high amounts to adsorb onto the walls of tubes decreasing, therefore, the possibility of less concentrated proteins, as amyloids, to stick to the plastic. This characteristic is commonly used in ELISA test by saturating the plastic wells with albumin, gelatin, or milk proteins after coating the capture antibody. Currently, when 1 h at 37°C is enough to saturate non-specific residual sites, it takes overnight at 4°C, what is absolutely consistent with the recommendation we did, to spin at low temperature.

Despite the generalized use of atraumatic needles, the influence of blood contamination is still relevant, and controversial results have been reported. Bjerke et al. observed that up to 5000 RBC/μL of CSF had no effect on Aβ42 levels (27). However, Zimmermann et al. showed that approximately 1 g/L of CSF protein levels (that can happen after traumatic tap or in patients with disrupted blood brain barrier) could have an impact on AD biomarkers (13). In our study, the inclusion of RBC count as a co-variate had no effect on biomarkers levels. However, the majority of our samples had low or just above threshold RBC counts, thus the influence of CSF contamination with RBC could not be ruled out. Spiking blood in CSF was tested for few samples, the final concentration of RBC being 5000/μL of CSF. Even if, they need to be confirmed, as the increase of levels was found under or close to the analytical CV of duplicates of 10%, our preliminary data are consistent with current guidelines in which it is recommended to centrifuge samples to avoid blood contamination. Further studies should be made with spiked CSF to clarify the influence of high RBC content on biomarkers quantification, using at least different amounts of spike, for example, in a range of RBC 5000–10,000/μL.

Aliquot storage volume is another potential pre-analytical confounder that has not been often assessed. We addressed this potential source of variation by storing three different ratios (25, 50, and 75%) of CSF volume per total tube capacity. The tube filling volume did not influence CSF levels of Tau and pTau. Concerning Aβ42 levels, they decreased when CSF storage volume decreased from 75 to 50% and amazingly, they slightly increased in 25% filled tubes, as compared to 50% filling. The variations were found lower than the accepted analytical intra-assay range-to-average of duplicates (<20%), so in our study using aliquots in tubes of 500 μL, the filling tube is not a strong confounder. It can be hypothesized that decreasing the ratio of CSF volume to surface area of storage tube would lead to an increased analyte adsorption to the internal walls of the tube, lowering its levels in solution. Our results are in accordance with a recent study testing the influence of a wide range of CSF volumes (2.5–75% of CSF per total tube volume) in Aβ42, Tau, and pTau measurements (31). While Tau and pTau remained stable with the increase in storage volume percentage, they found that a volume increase of 10 μL caused an Aβ42 increase of 1 pg/mL, which is absolutely consistent with the increase of 3.7% between 250 and 380 μL (13 pg/mL) that we have found. In the studies by Toombs and colleagues (28, 31), the addition of 0.05% Tween 20 to the aliquots resulted in considerably higher concentrations of Aβ42, suggesting that in the presence of detergent a higher proportion of Aβ42 molecules were free in solution, thus supporting the hypothesis of protein adsorption to the tube walls, as previously reported (27). This is consistent with the fact that Tween 20 is used as a blocking agent in ELISA plates, avoiding further adsorption of proteins.

The influence of freeze/thaw cycles (one, three, and five) during CSF storage, before protein measurements, was also investigated. We observed that Tau and pTau levels were not altered, but Aβ42 levels decreased slightly (5%) with repeated freeze/thawing, especially above three cycles, but as reported for filling tube study, this decrease was under the accepted analytical intra-assay range-to-average of duplicates (<20%); so, in our study, using three freeze/thaw cycles has no strong effect onto the CSF levels of biomarkers. Our data are consistent with those of Zimmerman et al. (13) reporting the stability for up to three freeze/thaw cycles for the three biomarkers and partially with those of Simonsen et al. (24) reporting stable levels for Aβ42 but increased levels for pTau without inhibitors of protease. Our data are mainly different with those obtained in the study of Schoonenboom et al. (30), in which they showed that after three freeze–thaw cycles (for 2 h) Aβ42 levels decreased by 20%, mainly after the first cycle whereas Tau protein was not altered by six freeze–thaw cycles. In this last study, the exact reference of PP tubes used for the study was not done, whereas in study of Simonsen et al., the tubes were exactly the same that those we selected as tubes known to present a minimal adsorption of amyloid. So, the difference between data concluding absence of strong effect onto amyloid levels and those showing large decrease of amyloid levels could be explained by a larger synergistic effect of adsorption and freeze/thaw process in studies in which the reference of tubes was not given and the adsorption of amyloid onto tubes was not checked (30). Therefore, most of the data obtained before the standardization of tubes must be interpreted with caution.

It should also be emphasized that, in our study, all measurements were carried out within 1 month storage and it cannot be ruled out that in samples kept for longer storage periods, different results could be obtained. We wanted to know to what extent the analysis of each condition would change after 1 and 2 years of storage. This was not performed after all, since it would surpass the length of the project. However, we would be reasonably comfortable to perform this analysis since there have been a few studies addressing the long-term stability of CSF biomarkers, concluding that Aβ42 and Tau proteins remain stable up to 6 years (if stored at −80°C immediately after collection and processing) (23), supporting the feasibility of biobanking over a large period of time. Thus, having this factor controlled, it would be possible to test variations within pre-analytical conditions.

In recent years, a strong effort has been done to develop and implement USOPs for CSF analysis, and this is also a major goal of the JPND-BIOMARKAPD Consortium. However, overall variability remains too high to allow assignment of universal biomarker cut-off values and is still compromising AD-like scores across laboratories (32–34). Taking this into account, our findings reinforce the existing guidelines and support new recommendations for CSF pre-analytical SOPs (Table 3).

**Table 3:** **Recommendations for CSF pre-analytical handling prior to evaluation of AD biomarkers**.

Confounder	Recommendation
Spinning	Right after collection (within 2 h max); at 2000 × *g* 4°C – avoid multiple tube transfers
Storage volume	Tubes should be filled up close to total tube capacity to keep constant the relation surface area/tube walls
Freeze–thaw cycles	Avoid more than three cycles to prevent protein degradation

We propose that centrifugation should be performed as fast as possible after CSF collection, at 4°C, the speed conditions being not a major factor (500, 2000, or 3000 × *g*); multiple tube transfer of CSF should be avoided and kept to a minimum. Storage aliquots should be filled up close to the maximum tube capacity in order to keep a constant surface area and avoid sublimation. It is preferable that samples should not be submitted to more than three freeze–thaw cycles to prevent protein degradation.

We strongly believe that this work will contribute to the establishment of core and broadly used feasible guidelines that will enable decisive AD large scale studies.

## Conflict of Interest Statement

The authors declare that the research was conducted in the absence of any commercial or financial relationships that could be construed as a potential conflict of interest.
